# CNS involvement in myotonic dystrophy type 1: does sex play a role?

**DOI:** 10.3389/fneur.2024.1399898

**Published:** 2024-05-09

**Authors:** Joana Garmendia, Garazi Labayru, Jone Aliri, Adolfo López de Munain, Andone Sistiaga

**Affiliations:** ^1^Department of Clinical and Health Psychology and Research Methodology, Psychology Faculty, University of the Basque Country (UPV/EHU), Donostia-San Sebastián, Gipuzkoa, Spain; ^2^Centro de Investigación Biomédica en Red sobre Enfermedades Neurodegenerativas (CIBERNED), Institute Carlos III, Madrid, Spain; ^3^Neuroscience Area, Biogipuzkoa Health Research Institute, Donostia-San Sebastián, Gipuzkoa, Spain; ^4^Neurology Department, Donostia University Hospital, Donostia-San Sebastián, Gipuzkoa, Spain; ^5^Neuroscience Department, University of the Basque Country (UPV/EHU), Donostia-San Sebastián, Gipuzkoa, Spain

**Keywords:** myotonic dystrophy type 1, DM1, sex, inheritance pattern, CTG, genomic imprinting, CNS, cognition

## Abstract

**Introduction:**

Myotonic dystrophy type 1 (DM1) is a hereditary neuromuscular disorder affecting the central nervous system (CNS). Although sex differences have been explored in other neuromuscular disorders, research on this topic in DM1 remains limited. The present study aims to analyze sex differences (both the patient’s and disease-transmitting parent’s sex) with a focus on CNS outcomes.

**Methods:**

Retrospective data from 146 non-congenital DM1 patients were analyzed, including clinical, molecular, neuropsychological, and neuroradiological data. Sex and inheritance pattern differences were analyzed using t-tests, and ANOVA analyses were conducted to address the interactions.

**Results:**

Overall, no significant sex differences were observed except in certain cognitive domains. However, individuals with maternal inheritance showed larger CTG expansion size, lower estimated IQs, and poorer performance on visual memory, executive functions, and language domains than those with paternal inheritance. Notably, IQ performance was independently influenced by inheritance pattern and CTG expansion.

**Discussion:**

This study is the first to delve into sex differences in DM1 with a focus on CNS outcomes. While the results revealed the absence of a sex-specific clinic-molecular profile, more substantial CNS differences were observed between patients with maternal and paternal inheritance patterns. The hypothetical existence of genomic imprinting and its potential mechanism are discussed. These findings hold potential implications for aiding clinical management by improving genetic counseling and predicting disease severity and prognosis.

## 1 Introduction

Myotonic dystrophy type 1 (DM1) is a multisystemic disease that affects various body systems, including muscular, ophthalmological, cardiac, endocrine, gastrointestinal, respiratory, and central nervous systems. This disorder is the most prevalent form of muscular dystrophy in adults, characterized by distal muscle weakness and myotonia, which patients typically present (1).

DM1 is a hereditary disease transmitted in an autosomal dominant manner and is diagnosed by detecting an expansion length of the trinucleotide CTG (cytosine, thymine, guanine) that exceeds 50 repetitions. This neuromuscular disorder is characterized by phenotypical variability in its clinical signs/symptoms and severity. However, it is well-established that CTG expansion size correlates positively with disease severity (1–3). Like other repeat expansion diseases, DM1 patients typically present a phenomenon known as clinical anticipation, which involves an earlier disease onset and more severe symptoms in successive generations of a family (4). Research has also investigated genomic imprinting in DM1, which consists of the differential phenotypic expression of genetic material depending on whether it has been inherited from the male or female parent (5). However, this line of inquiry is not currently being pursued. DM1 is commonly classified into different phenotypes based on the age of onset of the disease: congenital (present at birth), childhood (onset between1-10 years of age), juvenile (onset between 10–20 years of age), adult (onset between 20–40 years of age) and late-onset (onset >40 years) (6).

To date, limited research has explored sex differences within the various systems affected by DM1. Some previous studies have focused on ophthalmological aspects (7), endocrinal differences (8), as well as pain and motor function (9, 10). Additionally, more general studies have been conducted on sex differences in clinical conditions or comorbidities in DM1 (11, 12). The latter study has revealed that sex influences the clinical profile and severity of the disease in DM1 patients. While male patients tend to present higher morbidity and mortality, including more pronounced muscular, cardiac, and respiratory impairments, female patients are more prone to experiencing ophthalmological, gastrointestinal, and endocrine-related issues.

However, no studies have focused on sex differences regarding CNS involvement in DM1. In addition to considering the sex differences between patients, it is important to explore the implications of the sex of the disease-transmitting parent, known as the patient’s inheritance pattern, whether the aberrant gene has been inherited from the mother (maternal) or father (paternal).

The scientific literature highlights that maternal inheritance has been observed at a higher percentage in DM1 patients with the congenital form (6, 13, 14), considered the most severe DM1 phenotype; indeed, the literature recognizes it as a clinically different form (13, 15). Therefore, it is important to investigate whether patients with maternal inheritance present greater disease severity in different DM1 phenotypes beyond the congenital form.

To date, studies have examined the influence of the inheritance pattern on the molecular instability of the disease (CTG expansion size) (16). However, no studies have explored the direct implications of the inheritance pattern for disease severity, specifically CNS affectation.

The present study aims to (1) analyze sex differences in terms of CNS, (2) examine differences between patients with maternal and paternal inheritance in terms of CNS involvement, and (3) analyze the interactions between inheritance pattern and CTG in relation to CNS involvement.

## 2 Methodology

### 2.1 Participants

For this study, retrospective data from a cohort of 146 DM1 patients recruited at the Neurology Department of the Donostia University Hospital (Gipuzkoa, Spain) were analyzed. Inclusion criteria included a molecular confirmation of the disease and being aged 18 years or above. Exclusion criteria were having the congenital form of DM1 and a history of major psychiatric or somatic illness, acquired brain injury, or drug abuse.

This study was approved by the Ethics Committee for Clinical Investigation of the Health Department of Gipuzkoa (DMRM-2017-01), and all participants provided signed informed consent.

The data were compiled using the method outlined below and were analyzed retrospectively for this study.

### 2.2 Clinical data

Clinical data, such as phenotype and inheritance pattern, were obtained through the patients’ medical records. An estimated disease duration was derived from the range of age of onset and the age at assessment. An experienced neurologist recorded data on muscular impairment using the Muscular Impairment Rating Scale (MIRS).

### 2.3 Data on genetic determination

The participants’ cytosine thymine guanine (CTG) expansion size was obtained from clinical data. Genetic assessment was conducted using blood sample analysis (polymerase chain reaction in DMPK alleles up to approximately 100 CTG and southern blot analysis for larger expansions).

### 2.4 Neuropsychological data

Data on the neuropsychological performance of the participants was accessible. The assessment included IQ estimation and performance on six cognitive domains (attention/processing speed, verbal memory, visual memory, visuoconstruction, executive functions, and language). Table 1 shows the tests employed for IQ estimation and the cognitive domains assessed. These tests were administered by an experienced neuropsychologist blind to the clinical condition of the participants (i.e., disease form, inheritance pattern, CTG repeats, or MIRS).

**Table 1:** Tests employed for assessing IQ and performance in cognitive domains.

Cognitive domains	Tests
IQ estimation	Block Design (WAIS-III) (17) Vocabulary (WAIS-III) (17)
Attention/PS	Digit span (WAIS-III) (17) Stroop-word, Stroop-Color (18) CALCAP (Simple Reaction Time (RT), choice RT) (19) Corsi (WMS-III) (20)
Verbal memory	RAVLT: immediate, total, and delayed (21)
Visual memory	Rey-Osterrieth Complex Figure Test (delayed memory) (22)
Visuoconstruction	Block Design (WAIS-III) (17) Rey-Osterrieth Complex Figure Test (copy) (22)
Executive functions	Phonemic fluency (FAS) (23) Stroop (interference) (18) CALCAP (Sequential 1 RT, Sequential 2 RT) (19) TMT B (24)
Language	Vocabulary (WAIS-III) (17) Semantic fluency (23) BNT (25)

IQ, Intelligence Quotient; PS, Processing Speed; CALCALP, California Computerized Assessment Package; RAVLT, Rey Auditory Verbal Learning Test; TMT, Trail-Making Test; BNT, Boston Naming Test. Standardized T values of all tests were obtained according to Spanish population-based normative data. The mean T values were calculated for each cognitive domain.

### 2.5 Neuroradiological data

All magnetic resonance scans were conducted using a 1.5 T scanner (Achieva Nova, Philips). The current results were obtained from a high-resolution volumetric turbo field echo series (sagittal 3D T1 weighted acquisition, repetition time 7.2, echo time 3.3, flip angle 8, matrix 256 × 232, slice thickness 1 mm, voxel dimensions 1 × 1 × 1 mm, number of signal averages 1, no slices 160, gap 0, and a total scan duration 5 min 34 s).

Grey matter (GM) and White matter (WM) volumes were determined using voxel-based morphometry via the FMRIB Software Library (FSL version 6.01) (26). White matter lesions (WML) were assessed according to the Wahlund scale (27). When lesions >5 mm were identified, severity was rated from 0 (no lesions) to 3 (diffuse involvement).

### 2.6 Statistical analyses

Data were analyzed using the SPSS statistical package (version 27).

To explore sex differences for both patients and the transmitting parent (inheritance pattern), contingency analysis (chi-square) was used for categorical data, while parametric tests (*t*-test) were employed for interval data. Effect sizes were reported using Cohen’s *d,* categorized as small (*d* ≤ 0.49), moderate (*d* = 0.50–0.79), or high (*d* ≥ 0.80).

In addition, given the well-established association between inheritance pattern and CTG expansion size and the correlation between CTG and CNS involvement, an ANOVA was conducted. This analysis aimed to examine the independent effects of these two variables (CTG and inheritance pattern) and to evaluate the impact of their interaction on the CNS variables (cognitive and structural brain outcomes) under study.

## 3 Results

### 3.1 Participants

The retrospective data analysis included 146 DM1 patients. Table 2 illustrates the distribution of sex, inheritance pattern, and phenotype among these patients. The sample is equally distributed in terms of sex (50/50), with a notable prevalence of paternal transmission. Regarding phenotype, most patients were adult-onset DM1 patients, followed by juvenile-onset, late/partial-onset, and finally, childhood onset.

**Table 2:** Sex, inheritance pattern, and phenotype of the DM1 participants.

	n/n (%)
*Total*	146
*Sex*	
Female	73 (50%)
Male	73 (50%)
*Inheritance*	
Maternal	38 (28.1%)
Paternal	96 (71.1%)
Both	1 (0.7%)
*Phenotype*	
Childhood	11 (7.5%)
Juvenile	28 (19.2%)
Adult	89 (61.0%)
Late/partial	18 (12.3%)

### 3.2 Sex differences in DM1

Clinical, cognitive, and structural brain data were used to explore differences between female and male patients, and the findings are summarized in Table 3.

**Table 3:** Sex differences in clinical, cognitive, and brain structure outcomes.

Sex		Female	Male	Female *vs* Male		
	*n*	*M* (SD)	*M* (SD)	*t*	*p*	*Cohen’s d*
Age	145	42.78 (12.21)	43.36 (11.45)	0.30	0.768	0.05
CTG	139	651.46 (501.84)	550.84 (413.94)	−1.29	0.200	0.22
MIRS	125	2.84 (0.16)	2.75 (0.13)	−0.46	0.649	0.08
Disease duration	145	16.91 (9.29)	14.61 (7.27)	−1.66	0.100	0.27
Years of education	143	13.97 (4.32)	13.87 (4.64)	−0.13	0.895	0.02
Estimated IQ	139	89.33 (15.22)	90.39 (15.38)	0.41	0.689	0.07
Attention/PS	144	40.10 (9.13)	43.35 (9.39)	2.10	**0.037** ^*^	0.35
Visual Memory	133	41.26 (10.90)	44.96 (9.78)	2.06	**0.042** ^*^	0.36
Verbal Memory	141	48.04 (10.40)	44.28 (10.83)	−2.10	**0.038** ^*^	0.35
Visuoconstruction	141	41.16 (10.15)	42.27 (8.39)	0.71	0.477	0.12
Executive functions	140	41.16 (9.06)	43.37 (10.00)	1.34	0.182	0.23
Language	144	46.69 (8.67)	47.17 (9.06)	0.32	0.746	0.05
GM	21	744927.35 (61664.56)	744024.23 (39049.67)	−0.04	0.969	0.02
WM	21	671875.13 (54002.78)	695882.77 (39777.83)	1.15	0.265	**0.50**
WML	12	2.00 (1.79)	7.17 (6.15)	1.98	0.097	**1.14**

M, Mean; SD, Standard Deviation; CTG, Cytosine Thymine Guanine expansion size; MIRS, Muscular Impairment Rating Scale; IQ, Intelligence Quotient; PS, Processing Speed; GM, Grey Matter Volume; WM, White Matter Volume; WML, White Matter Lesions. ^*^p < 0.05. Bold values in the *p*-values, is the statistical significance, and in Cohen’s d a moderate to high effect size.

Regarding clinical data, both female and male patients presented a similar phenotype distribution (*χ*^2^ (1) = 1.28; *p* = 0.733), and no sex differences were found in CTG, MIRS, and disease duration among DM1 patients.

In terms of cognitive performance, female patients showed significantly poorer performance in the attention/processing speed and visual memory domain. In contrast, male patients performed significantly worse in the verbal memory domain. However, the effect size was small to moderate in all cases.

Concerning structural brain outcomes, although not statistically significant, female patients tended to have lower WM volume, while male patients more WML, with moderate and high effect sizes, respectively.

### 3.3 Differences regarding inheritance pattern in DM1

Differences between patients with maternal and paternal inheritance patterns are summarized in Table 4. Notably, the patient with inheritance transmission from both parents was excluded from further analysis.

**Table 4:** Differences in clinical, cognitive, and brain structure outcomes according to inheritance pattern.

Inheritance pattern		Maternal	Paternal	Maternal *vs* Paternal		
	*n*	M (SD)	M (SD)	*t*	*p*	*Cohen’s d*
Age	133	38.68 (10.78)	42.86 (11.02)	1.97	0.051	0.38
CTG	128	836.24 (595.40)	542.22 (353.99)	−2.81	**0.007** ^**^	**0.67**
MIRS	114	2.85 (1.06)	2.86 (1.08)	0.07	0.944	0.01
Disease duration	133	15.82 (8.49)	15.59 (8.12)	−0.14	0.888	0.03
Years of education	131	13.11 (4.32)	14.36 (4.30)	1.47	0.144	0.29
Estimated IQ	129	82.42 (16.82)	91.87 (13.65)	3.30	**0.001** ^**^	**0.65**
Attention/PS	132	39.81 (8.99)	42.26 (8.76)	1.68	0.095	0.33
Visual Memory	123	39.81 (9.81)	43.98 (10.77)	2.00	**0.047** ^*^	0.40
Verbal Memory	130	45.14 (8.22)	46.57 (11.73)	0.78	0.436	0.13
Visuoconstruction	130	39.51 (10.71)	42.15 (8.87)	1.43	0.155	0.28
Executive functions	129	38.86 (9.38)	43.36 (10.26)	2.31	**0.023** ^*^	**0.45**
Language	132	42.49 (8.69)	48.39 (8.48)	3.57	**0.001** ^**^	**0.69**
GM	20	745095.05 (72702.25)	742833.18 (30442.84)	−0.09	0.926	0.04
WM	20	686348.57 (56455.06)	676028.81 (41812.25)	−0.47	0.644	0.21
WML	11	2.50 (1.91)	6.14 (6.23)	1.43	0.191	**0.70**

M, Mean; SD, Standard Deviation; CTG, Cytosine Thymine Guanine expansion size; MIRS, Muscular Impairment Rating Scale; IQ, Intelligence Quotient; PS, Processing Speed; GM, Grey Matter Volume; WM, White Matter Volume; WML, White Matter Lesions. ^*^p < 0.05; ^**^p < 0.01. Bold values in the *p*-values, is the statistical significance, and in Cohen’s d a moderate to high effect size.

Regarding clinical data, there was a significant difference in phenotype distribution between maternal and paternal inheritance patients (*χ*^2^ (1) = 39.07; *p* < 0.001). Specifically, childhood and juvenile-onset phenotypes were more prevalent among patients with maternal inheritance, while those with paternal inheritance presented more adult and late-onset phenotypes.

Furthermore, patients with maternal inheritance showed significantly larger CTG expansion (moderate-high effect size). However, no significant muscular and disease duration differences were observed between maternal and paternal inheritance.

In relation to cognition, as shown in Table 4, DM1 patients with maternal inheritance demonstrated poorer cognitive performance than those with paternal inheritance. Specifically, when examining statistically significant differences, DM1 patients with maternal inheritance presented a lower estimated IQ and exhibited inferior performance in the cognitive domains of visual memory, executive functions, and language (moderate-high effect sizes) compared to patients with paternal inheritance.

In terms of brain structure, volume differences were not statistically significant. However, there was a moderate to high effect size in the difference between patients with paternal and maternal inheritance in WML, with paternal inheritance patients showing more lesions.

### 3.4 Analysis of interaction between inheritance pattern and CTG for CNS variables

Upon further analysis of the differences in CNS outcomes between maternal and paternal inheritance, the following results emerged.

**Figure 1:**
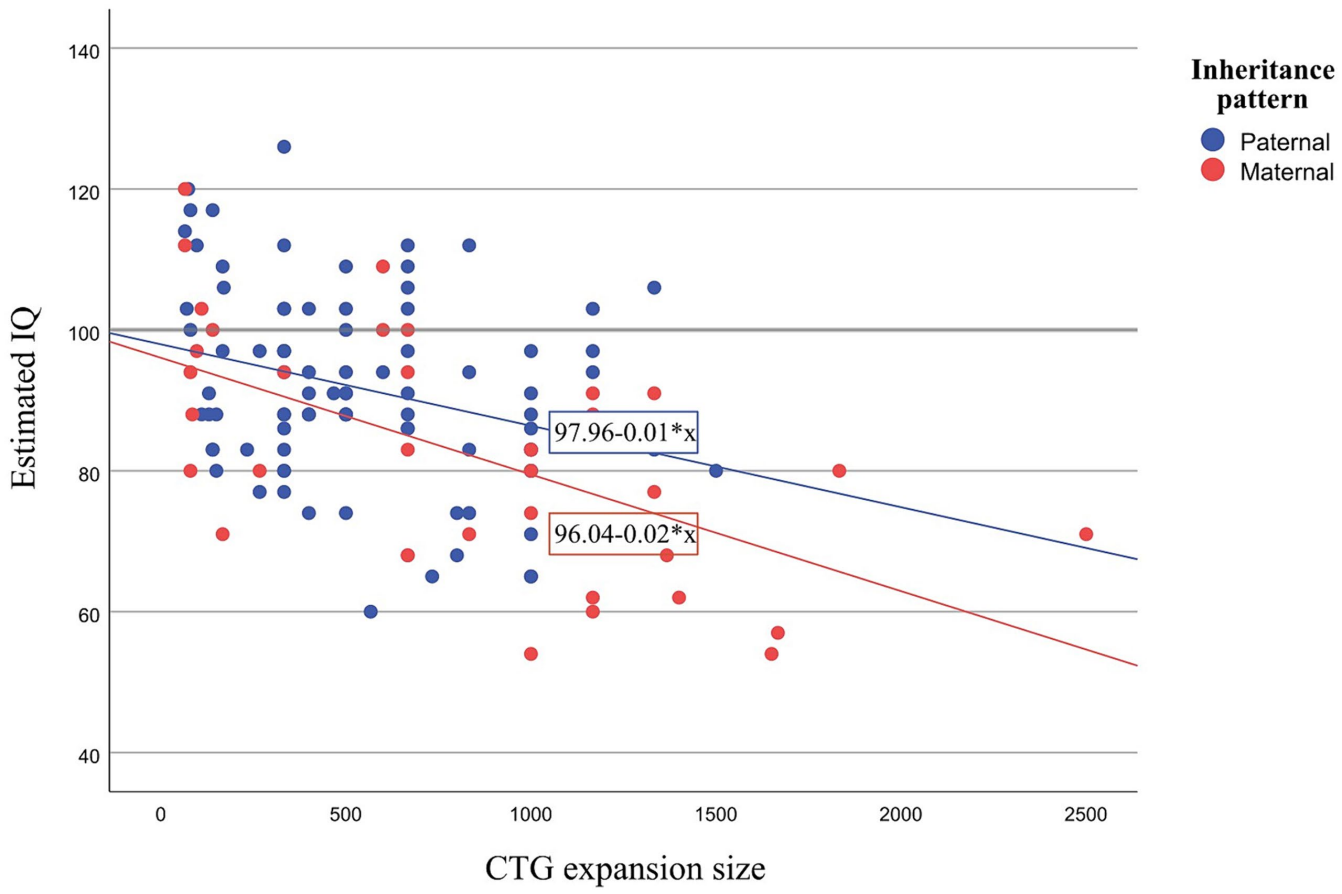
Scatter plot showing estimated IQ and CTG for paternal and maternal inheritance. IQ, Intelligence Quotient; CTG, Cytosine Thymine Guanine.

Notably, the reported differences in IQ were independently influenced by both the inheritance pattern and CTG expansion size (independent effect), with no interaction between these variables. Specifically, maternal inheritance and larger CTG expansion size were associated with a lower IQ estimation. To better illustrate the independent effects of inheritance pattern and CTG expansion size on estimated IQ, a graph is displayed in Figure 1. This graph shows that patients with maternal inheritance typically present lower IQ and that the effect of CTG is greater than in paternal inheritance. This finding implies that for the same CTG expansion size, for example, a CTG expansion size of 300, the expected IQ in a patient with maternal inheritance would be 90.04, whereas in a paternal inheritance patient, it would be 94.96 (Maternal inheritance: 96.04–0.02*300 = 90.04; Paternal inheritance: 97.96–0.01 * 300 = 94.96).

Analysis of the remaining cognitive domains revealed an interaction between inheritance pattern and CTG in the visual memory domain. Additionally, an independent effect of inheritance pattern was observed in the language domain, with CTG not emerging as a significant predictor. Finally, no significant differences were reported for executive functioning, which could potentially be attributed to CTG being controlled or the lower statistical power in this analysis due to the inclusion of more variables.

The following table (Table 5) displays the results of ANOVA analyses for CNS variables where differences were identified between maternal and paternal inheritance.

**Table 5:** ANOVA showing the effect of inheritance pattern, CTG and their interaction over cognitive performance.

	IQ		Visual memory		Executive functioning		Language	
Statistics	*F*	*p*	*F*	*p*	*F*	*p*	*F*	*p*
Inheritance pattern	5.54	0.021^*^	0.01	0.940	0.89	0.349	6.26	0.014^*^
CTG	2.60	<0.001^**^	0.97	0.525	1.51	0.073	1.12	0.336
Interaction	1.07	0.401	1.98	0.035^*^	0.89	0.574	1.70	0.076

CTG, Cytosine Thymine Guanine expansion size; Interaction, Inheritance pattern x CTG. ^*^p < 0.05; ^**^p < 0.01.

## 4 Discussion

Sex differences have been studied in neuromuscular disorders, shedding light on potential differences in prevalence, onset, severity, and clinical progression of the disease between male and female patients (28). However, there is currently limited literature addressing sex differences in DM1. This study represents the first investigation focusing on sex differences in CNS involvement within this population.

Overall, the main finding of this study indicates that male and female DM1 patients show a similar clinical profile, as assessed by the variables examined in this study. Only subtle differences were found in certain cognitive domains and brain outcomes. A previous study exploring sex differences across a broad spectrum of clinical signs/symptoms in DM1 (11) indirectly assessed cognitive performance based on educational level and suggested that male patients presented more cognitive impairment than females. However, this result was not obtained in this study, in which a comprehensive cognitive assessment was conducted.

Regarding the sex of the transmitting parent (inheritance pattern), this study confirmed that the phenotype distribution differed between both groups. Specifically, patients who inherited the disease from their mother presented an earlier onset of signs and symptoms (childhood and juvenile-onset DM1), while those who inherited it from their father presented a later onset of signs and symptoms (adult and late-onset DM1). However, some authors have suggested that inheritance pattern does not impact the development of childhood-onset DM1 (6, 14). Despite earlier disease onset observed in maternally inherited patient in this study, there was no significant difference in disease duration between individuals with maternal and paternal inheritance. This might be attributed to the slight age difference, although not statistically significant, with maternally inherited patients being slightly younger.

At a molecular level, patients with maternal inheritance showed greater molecular instability (CTG expansion size) than paternally inherited patients, which could also explain the earlier onset of symptoms mentioned previously, considering that CTG typically correlates with disease severity and age of onset. Similarly, another study reported that the sex of the transmitting parent might determine the molecular defect of their offspring who inherited DM1 (16).

While maternal inheritance DM1 patients showed a greater molecular defect, they were no more muscularly affected than patients with paternal inheritance. This result could be due to the measure employed to address muscular impairment. Although the MIRS is commonly employed for this purpose, the limited range of the scale could hinder the discrimination of patients’ muscular variability.

In terms of CNS outcomes, DM1 patients with maternal inheritance presented lower IQ scores and poorer performance across various cognitive domains. It is worth noting the effect of CTG expansion size on these differences in cognitive performance between maternal and paternal inheritance patients. However, this study confirms that inheritance pattern affects certain cognitive domains regardless of CTG expansion size. For instance, when comparing two patients with identical CTG expansion sizes, the estimated IQ of the patient with maternal inheritance is expected to be lower than that of the patient with paternal inheritance.

Although recent literature has questioned the phenomenon of genomic imprinting in DM1 (13), this study suggests that the sex of the transmitting parent has phenomic significance, resulting in a differential clinical profile.

These findings open the possibility of the existence of a factor linked to the gestation period within the uterine environment of a DM1 mother, which could affect the future cognitive development of the DM1 siblings independently of the CTG size. During the premolecular stage of DM1 research, several authors speculated about the presence of a humoral factor to explain the fact that nearly all patients with congenital forms were born from affected mothers (29, 30). With the discovery of the molecular substrate of the disease and the intergenerational increase in expansion size differing by the sex of the transmitter, this hypothesis was disregarded.

However, the data from this study would allow a reconsideration of the hypothesis regarding the existence of this specific gestational factor. This factor could exhibit an epigenetic character, associated with specific changes in the function of certain genes due to processes of differential methylation or even a distinct proportion and biological effect of circulating miRNAs (31). Another non-exclusive possibility to the aforementioned is that alternative splicing of unknown maternal genes could induce aberrant proteins that traverse the placenta, generating changes in fetal brain development. Future studies with large DM1 cohorts and/or experimental studies in animal models, would be necessary to explore the existence and impact of this hypothetical maternal factor.

This study is not free of limitations. One of the main constraints when studying a rare condition is the sample size, although the sample recruited for this study was considerable. Nevertheless, not all participants underwent MRI scanning, resulting in a smaller sample size for assessing brain structural outcomes. Another issue worth considering is the high percentage of paternal inheritance (71.1%) observed in the DM1 sample, which should be taken into account when generalizing the results to the entire DM1 population. However, this higher percentage of paternal inheritance is a trend commonly observed in the literature, with reported rates of paternal transmission ranging from 42–50% for infantile DM1, 68–72% for juvenile DM1, and approximately 70% for adult and late-onset DM1 (11, 32). Furthermore, it is worth noting that congenital DM1 patients — who typically present maternal inheritance — were excluded from this study, which could partially explain the lower percentage of maternal transmission observed. Finally, the long-established genetic counseling provided by the Neurology Service in our region might have influenced the decrease in cases of maternally transmitted DM1.

Overall, does sex play a role in DM1? In general terms, while the clinic-molecular and CNS profiles of males and females with DM1 appear to be similar, the results of this study suggest that the inheritance pattern influences CNS outcomes, with a poorer prognosis observed for those with maternal inheritance. Therefore, regarding CNS involvement, sex appears to play a role, but only in relation to the sex of the transmitting parent.

The implications of these findings should be considered in clinical practice, particularly in genetic counseling and when informing patients about disease severity and prognosis. Ultimately, this knowledge can help to tailor healthcare management to meet the specific needs of each patient.

## Data availability statement

The original contributions presented in the study are publicly available. This data can be found here: https://osf.io/pzgxh.

## Ethics statement

The studies involving humans were approved by Ethics Committee for Clinical Investigation of the Health Department of Gipuzkoa (DMRM-2017-01). The studies were conducted in accordance with the local legislation and institutional requirements. The participants provided their written informed consent to participate in this study.

## Author contributions

JG: Conceptualization, Data curation, Formal analysis, Methodology, Visualization, Writing – original draft, Writing – review & editing. GL: Conceptualization, Investigation, Methodology, Supervision, Validation, Writing – review & editing. JA: Data curation, Formal analysis, Supervision, Writing – review & editing. AM: Investigation, Resources, Supervision, Validation, Writing – review & editing, Conceptualization, Project administration. AS: Conceptualization, Funding acquisition, Investigation, Project administration, Resources, Supervision, Validation, Writing – review & editing.

## References

[1] Harper P. Myotonic dystrophy. 3rd ed. London, UK: Saunders (2001).

[2] Bigot A Klein AF Gasnier E Jacquemin V Ravassard P Butler-Browne G. Large CTG repeats trigger p16-dependent premature senescence in myotonic dystrophy type 1 muscle precursor cells. Am J Pathol. (2009) 174:1435–42. doi: 10.2353/ajpath.2009.080560, PMID: 19246640 PMC2671374

[3] Hunter A Tsilfidis C Mettler G Jacob P Mahadevan M Surh L. The correlation of age of onset with CTG trinucleotide repeat amplification in myotonic dystrophy. J Med Genet. (1992) 29:774–9. doi: 10.1136/jmg.29.11.774, PMID: 1453425 PMC1016169

[4] Paulson H. Repeat expansion diseases. Handbook Clin Neurol. (2018) 147:105–23. doi: 10.1016/B978-0-444-63233-3.00009-9, PMID: 29325606 PMC6485936

[5] Chatkupt S Antonowicz M Johnson WG. Parents do matter: genomic imprinting and parental sex effects in neurological disorders. J Neurol Sci. (1995) 130:1–10. doi: 10.1016/0022-510X(94)00284-U, PMID: 7650524

[6] Turner C Hilton-Jones D. The myotonic dystrophies: diagnosis and management. J Neurol Neurosurg Psychiatry. (2010) 81:358–67. doi: 10.1136/jnnp.2008.158261 20176601

[7] Scutifero M Lanza M Petillo R De Bernardo M Passamano L Rosa N. Gender effect on onset, prevalence and surgical treatment of cataract in patients with myotonic dystrophy type 1. Acta Myol. (2022) 41:105–10. doi: 10.36185/2532-1900-N75, PMID: 36349183 PMC9628803

[8] Spaziani M Semeraro A Bucci E Rossi F Garibaldi M Papassifachis MA. Hormonal and metabolic gender differences in a cohort of myotonic dystrophy type 1 subjects: a retrospective, case–control study. J Endocrinol Investig. (2020) 43:663–75. doi: 10.1007/s40618-019-01156-w, PMID: 31786795

[9] Liguori S Moretti A Toro G Paoletta M Palomba A Barra G. Pain and motor function in myotonic dystrophy type 1: a cross-sectional study. IJERPH. (2023) 20:5244. doi: 10.3390/ijerph20075244, PMID: 37047859 PMC10094252

[10] Solbakken G Løseth S Froholdt A Eikeland TD Nærland T Frich JC. Pain in adult myotonic dystrophy type 1: relation to function and gender. BMC Neurol. (2021) 21:101. doi: 10.1186/s12883-021-02124-9, PMID: 33663406 PMC7931522

[11] Dogan C De Antonio M Hamroun D Varet H Fabbro M Rougier F. Gender as a modifying factor influencing myotonic dystrophy type 1 phenotype severity and mortality: a Nationwide multiple databases cross-sectional observational study. PLoS One. (2016) 11:e0148264. doi: 10.1371/journal.pone.0148264 26849574 PMC4744025

[12] Ouyang L Wang Y Valdez R Johnson N Gutmann L Street N. Gender difference in clinical conditions among hospitalized adults with myotonic dystrophy. Muscle Nerve. (2019) 59:348–53. doi: 10.1002/mus.26402, PMID: 30575975 PMC7702279

[13] Lanni S Pearson CE. Molecular genetics of congenital myotonic dystrophy. Neurobiol Dis. (2019) 132:104533. doi: 10.1016/j.nbd.2019.104533 31326502

[14] Stokes M Varughese N Iannaccone S Castro D. Clinical and genetic characteristics of childhood-onset myotonic dystrophy. Muscle Nerve. (2019) 60:732–8. doi: 10.1002/mus.26716, PMID: 31520483

[15] Meola G Cardani R. Myotonic dystrophies: an update on clinical aspects, genetic, pathology, and molecular pathomechanisms. Biochim Biophys Acta (BBA) - Mol Basis Dis. (2015) 1852:594–606. doi: 10.1016/j.bbadis.2014.05.019, PMID: 24882752

[16] Brunner HG Brüggenwirth HT Nillesen W Jansen G Hamel BC Hoppe RL. Influence of sex of the transmitting parent as well as of parental allele size on the CTG expansion in myotonic dystrophy (DM). Am J Hum Genet. (1993) 53:1016–23. PMID: 8213829 PMC1682295

[17] Wechsler D. WAIS-III: Escala de Inteligencia de Wechsler para Adultos III. Madrid: TEA Ediciones (1999).

[18] Golden CJ. Stroop color and word test. 3rd ed. Madrid: TEA Ediciones (2001).

[19] Miller EN. CalCAP: California computerized assessment package. Los Angeles: Norland Software (1990).

[20] Tulsky DS Chiaravalloti ND Palmer BW Chelune GJ. The Wechsler memory scale In: Clinical interpretation of the WAIS-III and WMS-III. 3rd ed. San Diego: Academic Press (2003). 93–139.

[21] Lezak M Howieson H Loring D. Neuropsychological assessment. 4th ed. New York: Oxford University Press (2004).

[22] Osterrieth PA. Le test de copie d’une figure complexe; contribution à l’étude de la perception et de la mémoire [Test of copying a complex figure; contribution to the study of perception and memory]. Arch Psychol. (1944) 30:206–356.

[23] Casals-Coll M Sánchez-Benavides G Quintana M Manero RM Rognoni T Calvo L. Estudios normativos españoles en población adulta joven (proyecto NEURONORMA jóvenes): normas para los test de fluencia verbal. Neurologia. (2013) 28:33–40. doi: 10.1016/j.nrl.2012.02.010, PMID: 22652141

[24] Pena-Casanova J Quinones-Ubeda S Quintana-Aparicio M Aguilar M Badenes D Molinuevo JL. Spanish multicenter normative studies (NEURONORMA project): norms for verbal span, visuospatial span, letter and number sequencing, trail making test, and symbol digit modalities test. Arch Clin Neuropsychol. (2009) 24:321–41. doi: 10.1093/arclin/acp038, PMID: 19661109

[25] Pena-Casanova J Quinones-Ubeda S Gramunt-Fombuena N Aguilar M Casas L Molinuevo JL. Spanish multicenter normative studies (NEURONORMA project): norms for Boston naming test and token test. Arch Clin Neuropsychol. (2009) 24:343–54. doi: 10.1093/arclin/acp039 19648582

[26] Andersson JL Jenkinson M Smith S. Non-linear registration aka spatial normalisation FMRIB Technial report TR07JA2. Oxford: FMRIB Analysis Group of the University of Oxford (2007).

[27] Wahlund LO Barkhof F Fazekas F Bronge L Augustin M Sjögren M. A new rating scale for age-related white matter changes applicable to MRI and CT. Stroke. (2001) 32:1318–22. doi: 10.1161/01.STR.32.6.1318, PMID: 11387493

[28] Vinciguerra C Iacono S Bevilacqua L Landolfi A Piscosquito G Ginanneschi F. Sex differences in neuromuscular disorders. Mech Ageing Dev. (2023) 211:111793. doi: 10.1016/j.mad.2023.111793 36806604

[29] Farkas-Bargeton E Barbet JP Dancea S Wehrle R Checouri A Dulac O. Immaturity of muscle fibers in the congenital form of myotonic dystrophy: its consequences and its origin. J Neurol Sci. (1988) 83:145–59. doi: 10.1016/0022-510X(88)90064-0, PMID: 3356987

[30] Harper P Dyken P. Early-onset dystrophia myotonica evidence supporting a maternal environmental factor. Lancet. (1972) 300:53–5. doi: 10.1016/S0140-6736(72)91548-6 4113301

[31] Perfetti A Greco S Cardani R Fossati B Cuomo G Valaperta R. Validation of plasma microRNAs as biomarkers for myotonic dystrophy type 1. Sci Rep. (2016) 6:38174. doi: 10.1038/srep38174, PMID: 27905532 PMC5131283

[32] Lagrue E Dogan C De Antonio M Audic F Bach N Barnerias C. A large multicenter study of pediatric myotonic dystrophy type 1 for evidence-based management. Neurology. (2019) 92:e852–65. doi: 10.1212/WNL.0000000000006948, PMID: 30659139

